# Medicine and Pauperism in Country Districts

**Published:** 1895-10-19

**Authors:** 


### SOCIAL PROBLEMS.

### MEDICINE AND PAUPERISM IN COUNTRY DISTRICTS.

The extent to which outdoor medical relief tends to sap the independence of the working classes is a matter of grave concern to the nation. Since the new Poor Law of 1833 laid down the principle that parish relief could not, except under very special circumstances, be distributed to persons in their own homes without producing incalculable evil results, a struggle has been going on between the advocates for indoor and outdoor relief which is ending in the almost universal discredit of the latter. But one loophole remains for evading the stern order to "come in," found so salutary in result and so painful to many authorities in practice. This loophole is medical relief. Mr. Chance, in his recent criticism of Poor Law administration,* points out that through this unsuspected channel a vast amount of ill-bestowed out-relief filters through in many lax unions, and that in the unions where a strict watch is kept in this particular, medical benefit clubs flourish and pauperism declines. The common feeling both of the guardians and of the public is that sickness breaks down all ordinary rules. If a man is ill, they argue, he must of course be doctored, and he has a right to choose whether it shall be at home or in an infirmary. Probably he will not make a good recovery unless he is better fed than usual, so that some measure of relief in kind must accompany the gift of medical advice. Moreover, the guardians are encouraged in laxity by the fact that in nearly all unions medical officers are paid by a fixed salary, so that the parish incurs no extra expenditure by his free attendance on any number of applicants.

The results, however, must be faced. The result which stands first in importance, viz., the lowering of the moral tone of the population, is a very difficult thing to gauge. Yet evidences are not wanting that in many country districts thrift has been seriously handicapped by the ease with which the doctor may be had for nothing, and without even the loss of self-respect and sensation of pauperism entailed by compulsory appearance before the Board. "The medical officer is frequently the only practitioner in the neighbourhood, so they see no manifest difference whether they lay by for illness, and then pay the doctor out of their own pocket, or whether they do not do this and yet are treated by the same person in the same way."† Yet the moral effects of these two courses lie poles asunder.

As to that form of medical relief to which grants of beef and brandy are annexed, it opens the door to more easily ascertainable abuses. Two instances are quoted by Mr. Chance as typical of frequent occurrences in connection with this form of grant; "one in which brandy destined for a pauper was consumed by the nurse in attendance, who was found by the neighbours, when they ultimately gained admittance to the room by forcing the door, lying in a state of intoxication across the body of the dying patient," and another where beef supplied for making beef tea for a sick person was found in the frying-pan preparing to make a good meal for the family. In addition to the difficulty of ensuring that the patient really benefits from the relief assigned to him is the encouragement given by Poor Law relief tickets to "malingering." The committee of the Brixham Board of Guardians report: "A very large proportion of cases now on the outdoor relief-list obtained relief in the first instance through medical certificates for some illness either feigned, or of a temporary nature, and have now become reduced to a state of almost hopeless pauperism." Cases of "debility" are reported as very common steps to outdoor relief in lax unions, and are regarded with suspicion by outside authorities. Pauperism is, indeed, in itself a disease of a most insidious and contagious description, and, like many other diseases, can be subdued only by firm measures and kept in check only by unrelaxing watchfulness.

Various remedies are proposed. The system in use in Bradfield (Berkshire), and one or two other unions, of granting medical relief on loan, has been found to work well. The medical officers, in addition to their salary, receive six shillings for each medical order granted on loan and recovered by the guardians from the applicant. It tends to diminish the number of unnecessary applications, and, of course, steps are taken to enforce payment only when the guardians are satisfied that there is ability to pay. The natural result of such action on the part of the parochial authorities is, that nearly every one belongs to a medical club, the payment being no more than four or five shillings a year for an adult, and two shillings for a child, with a maximum of twelve shillings for the family.

Other measures advocated by Mr. Chance for preventing abuses, are the enforcement of the rule that each applicant for parish relief shall attend the meeting of the Board of Guardians; the preparation of stimulants granted in out-relief, in such a way as to render them unpalatable; and the granting of medical extras only after strict inquiry, and for limited periods. A very important feature in restricting out-medical relief in country districts is the improvement of the accommodation in workhouse infirmaries, where alone the sick poor can be satisfactorily relieved. So long as pauper nurses are left in sole charge of the wards, and cases of old-age infirmity fill the wards—to the exclusion of cases of acute illness—the guardians are powerless to fall back on the only real alternative to outdoor medical relief among the very poor. Better buildings, more generous treatment, and skilled nursing will speedily convince the sick in country districts—as they have been convinced in London—that the infirmary is a far more satisfactory refuge in the hour of illness than the dole of the relieving officer, while the danger of imposture under treatment is by this means reduced to a minimum.

* "The Better Administration of the Poor Law." William Chance. (Charity Organisation Series.)

† Report of Local Government Board.

